# Energy-Efficient Integrated Circuit Solutions Toward Miniaturized Closed-Loop Neural Interface Systems

**DOI:** 10.3389/fnins.2021.667447

**Published:** 2021-05-31

**Authors:** Jaeouk Cho, Geunchang Seong, Yonghee Chang, Chul Kim

**Affiliations:** ^1^Biomedical Energy-Efficient Electronics Laboratory, Department of Bio and Brain Engineering, Korea Advanced Institute of Science and Technology, Daejeon, South Korea; ^2^KAIST Institute for Health Science and Technology, Daejeon, South Korea

**Keywords:** closed-loop system, neural interface, electroceuticals, ADC-direct front-end, miniaturization, stimulation artifact removal, wireless power transfer

## Abstract

Miniaturized implantable devices play a crucial role in neural interfaces by monitoring and modulating neural activities on the peripheral and central nervous systems. Research efforts toward a compact wireless closed-loop system stimulating the nerve automatically according to the user's condition have been maintained. These systems have several advantages over open-loop stimulation systems such as reduction in both power consumption and side effects of continuous stimulation. Furthermore, a compact and wireless device consuming low energy alleviates foreign body reactions and risk of frequent surgical operations. Unfortunately, however, the miniaturized closed-loop neural interface system induces several hardware design challenges such as neural activity recording with severe stimulation artifact, real-time stimulation artifact removal, and energy-efficient wireless power delivery. Here, we will review recent approaches toward the miniaturized closed-loop neural interface system with integrated circuit (IC) techniques.

## 1. Introduction

As life expectancy increases, the number of patients suffering degenerative brain diseases such as Parkinson's disease (PD) and Alzheimer's disease (AD) is rapidly increasing (Dorsey et al., 2007; Reitz et al., 2011). Several approaches including medication and surgery have been taken to tackle these degenerative brain diseases, and among them, the neural stimulation technique has proved its efficacy. Deep brain stimulation (DBS) has been widely used to suppress tremors of PD patients (Benabid et al., 1987), and the neural stimulation technique is effective to alleviate the symptoms of neurological diseases such as AD, depression, and epilepsy (Cook et al., 2014; Fox et al., 2014; Poewe et al., 2017; Chang et al., 2018). Furthermore, electroceuticals that control neural circuits using electrical pulses are recently gaining increasing interest from researchers (Famm et al., 2013, Levin et al., 2019).

**Figure 1:**
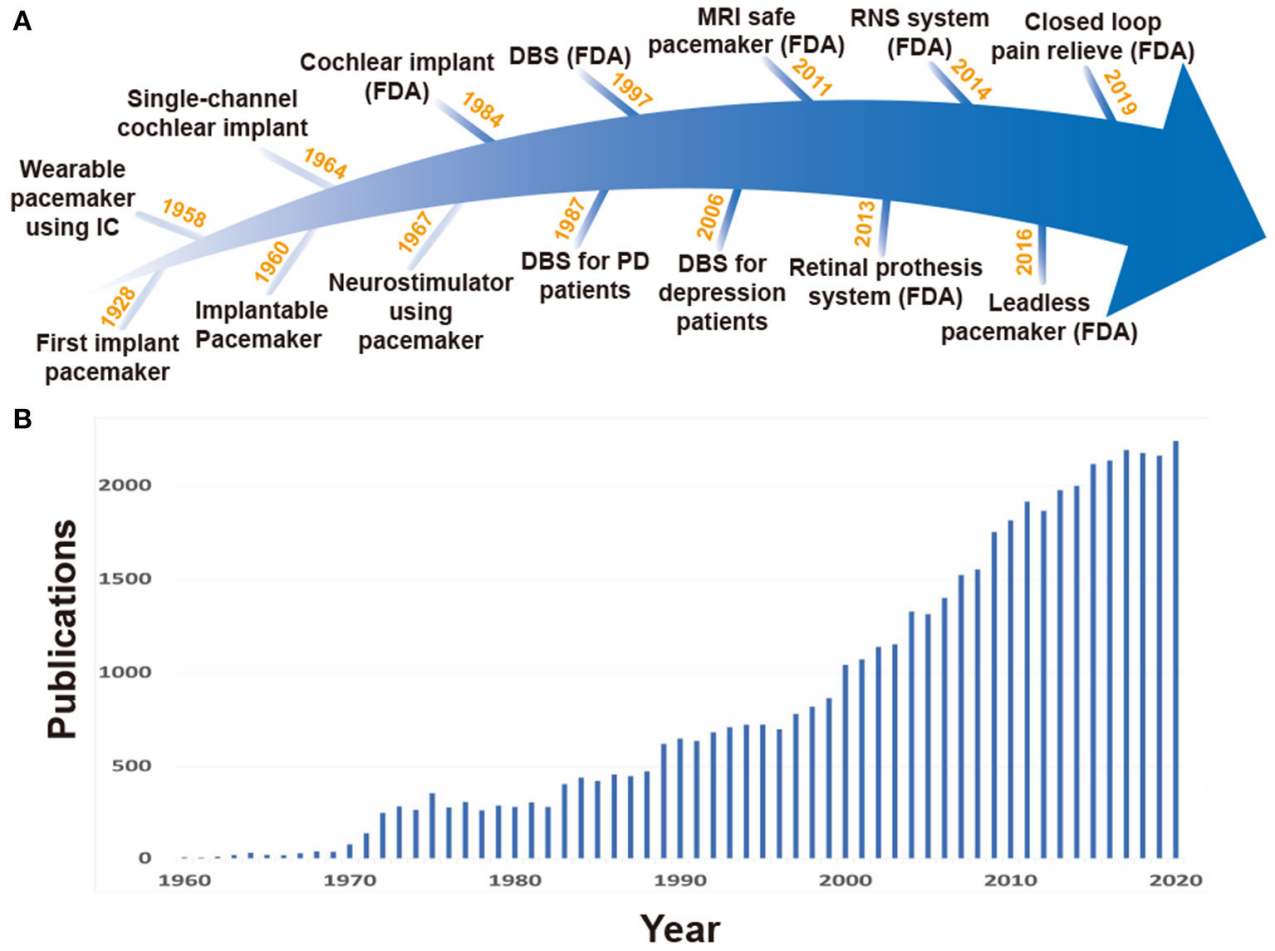
**(A)** History for stimulation devices. **(B)** Rapid increase in the number of publications on implantable stimulation devices, searched at PubMed (http://www.pubmed.gov).

Neural stimulation can be performed in either non-invasive or invasive fashions. Despite the disadvantage of requiring surgical operations, the invasive method is superior to the non-invasive one in specificity (Chen and Chen, 2019). The early stage of the invasive implantable neural stimulation devices began in 1928 with the pacemaker of Mark Lidwell (Aquilina, 2006), followed by the development of various stimulation devices, including cochlear implantation in 1964 and neurostimulator in 1967 as shown in Figure 1A (Simmons et al., 1964; Shealy et al., 1967).

The first generation of invasive stimulation devices have large forms due to large discrete components and battery size, and as a result, would be located outside of the body. Long wire connections between electrodes inside the body and such huge stimulation devices on the outside were required (Hyman, 1932). This led to severe restrictions on patient mobility. To enable normal life of the patients with such devices, studies of stimulation devices using IC technologies started. The use of IC technology was able to decrease overall device size by replacing large discrete components with miniaturized ICs. In 1958, the first wearable pacemaker device using IC technology was developed (Aquilina, 2006; Li et al., 2015), and in 1960, a fully implantable pacemaker with a battery was first applied to a human patient (Lillehei et al., 1960; Mallela et al., 2004). For better stimulation control and long operation time, various studies on multi-channel small form factor stimulation devices and wireless power transmission (WPT) have rapidly increased as shown in Figure 1B. Especially, features like closed-loop stimulation (responsive neural stimulation) measuring neural activities and performing stimulation only when necessary are attracting attention because they are able to lessen the side effects of open-loop stimulation (continuous stimulation) and further increase operation time given battery capacity (Bouthour et al., 2019). However, to enable closed-loop stimulation, neural activity recording and stimulation should be performed simultaneously, imposing serious challenges on circuit design.

In this paper, we review state-of-the-art implantable stimulation devices and requirements for implementing closed-loop electrical stimulation systems. In section 2, several neural stimulation devices using the modern technology are introduced. Various stimulation methods and considerations of stimulation designs are presented in section 3, while section 4 describes the requirements of the recording for the closed-loop system. Section 5 explains the origin of stimulation artifacts and techniques to alleviate them. Finally, various modalities for WPT to implants are reviewed in section 6, followed by conclusion in section 7.

## 2. Miniaturized Implants for Stimulation

**Figure 2:**
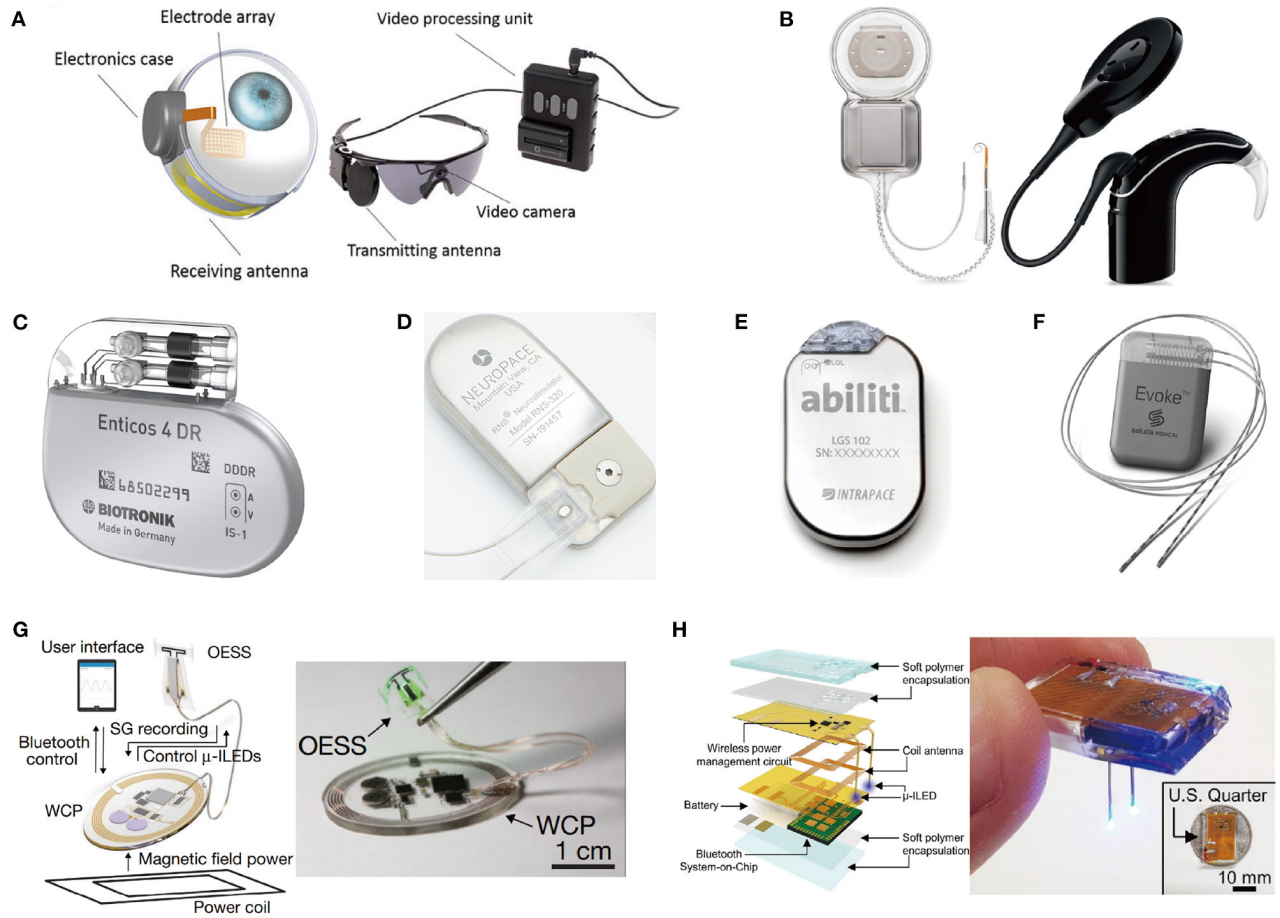
State of the art implant neural stimulation systems: **(A)** Argus II Retinal prosthesis implant operate (Farvardin et al., 2018); **(B)** Cochlear Contour^TM^ cochlear implant system (Parkinson et al., 2002); **(C)** Closed-loop pacemaker of Biotronik (Biotronik, aker); **(D)** Responsive neurostimulator (RNS) system of NeuroPace (Sun et al., 2008); **(E)** Abiliti closed-loop gastric stimulator (Horbach et al., 2015); **(F)** Evoke closed-loop spinal cord stimulation system (Russo et al., 2018); **(G)** Wireless optical peripheral stimulation system for bladder control (Mickle et al., 2019); **(H)** Fully chip-type implanted optical DBS system through the combination of wireless rechargeable battery and stimulator (Kim et al., 2021).

Treatment using drugs spreads throughout the entire body, which affects areas other than the desired target, and thus has a potential for side effects. On the other hand, stimulation therapy reduces side effects because the effects of stimulation spread locally (Famm et al., 2013). Stimulation therapy is also effective for people who have resistance to drug effects (Li and Cook, 2018). Therefore, neural stimulation devices have been developed for clinical and research purposes. In Figure 2, recent implantable stimulation devices are presented. Devices shown in Figures 2A,B have been developed for people whose vision or hearing cannot be treated by surgery or medication. These devices provide stimulation onto impaired parts and generate neural activity as if that impaired part operates as ordinary.

Most stimulation devices use a battery for a power source. However, regular surgery is needed to replace the battery in typically 5–10 years (Helmers et al., 2018; Sette et al., 2019). Periodic surgery increases the risk of infection as well as economic burden on patients (VanEpps and John G, 2016). The easiest solution to alleviate this periodic surgery issue is to adopt a large-capacity battery with large-sized battery. But, a large-sized battery causes protruding and reduces MRI compatibility (Belott, 2019). Besides, it may increase the size of the device, increasing the risk of tissue inflammation and cell death (McConnell et al., 2009; Karumbaiah et al., 2013). Some studies focused on rechargeable batteries using light that can be externally charged with a photovoltaic converter (Algora and Peña, 2009). Unfortunately, however, light cannot penetrate tissue deeply and charging efficiency is therefore poor, leading to rare use. Rather than focusing on battery capacity, reducing the energy consumption of stimulation devices is also a great alternative. Utilizing a closed-loop stimulation can significantly reduce power consumption compared to open-loop stimulation since power consumption of stimulation typically dominates that of the implant. Figures 2C–F shows commercialized closed-loop stimulation devices that perform responsive stimulation according to the patient's condition to increase the treatment effects and energy efficiency.

For certain applications, the battery size and weight of the closed-loop stimulation device are still too big and heavy to place near the stimulation site. Therefore, the stimulation device including batteries is needed to be placed apart from impaired parts. This results in long wire connections between electrodes and the device position as shown in Figure 2F. Implantation of this wire connection requires general anesthesia, and possibly causes lead dislodgement (Gul and Kayrak, 2011). Thus, research for miniaturization of implants has been actively conducted. The battery is a main culprit for the large size of implants. As such, researchers have recently been trying to use an extremely small-sized rechargeable battery or even trying to eliminate the battery as depicted in Figures 2G,H. This is possible since wireless power transmission via inductive or ultrasonic coupling became a main power source (Jow and Ghovanloo, 2007; Luo et al., 2013; Mickle et al., 2019).

## 3. Stimulation System Considerations

Stimulation systems have utilized various stimulation modalities such as electricity (Farvardin et al., 2018), light (Wells et al., 2005), temperature (Lee J. W. et al., 2018), and ultrasound (Norton, 2003; El-Bialy et al., 2011). Even in electrical stimulation, stimulation methods such as voltage-controlled stimulation (VCS), current-controlled stimulation (CCS), and switched-capacitor based stimulation (SCS) should be considered since stimulation methods affect stimulation safety and design complexity of stimulators. Furthermore, stimulation parameters such as stimulus duration, frequency, and waveform also have significant impacts on the efficacy of stimulation (Simpson and Ghovanloo, 2007; Wongsarnpigoon et al., 2010; Snellings and Grill, 2012).

### 3.1. Stimulation Modality

**Figure 3:**
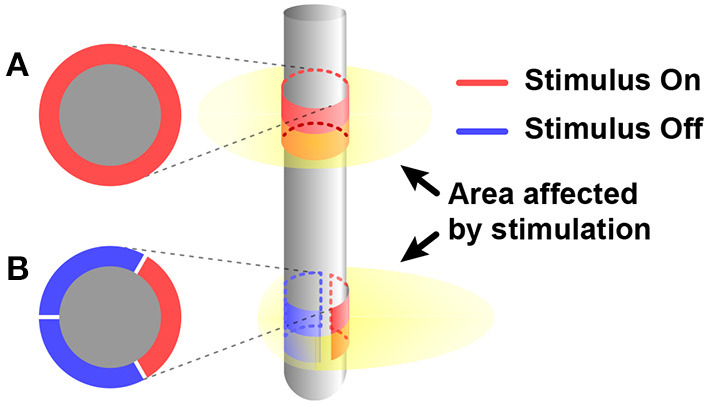
Comparing affected area by electrical stimulus between when using **(A)** conventional ring shape electrode and **(B)** directional electrode.

Since all nerves in a body are communicating in the form of electricity, electrical stimulation has potential to control a subject's body entirely (Famm et al., 2013). Furthermore, since injected charge can diffuse everywhere in tissue, electrical stimulation features its wide stimulation coverage. Therefore, electrical stimulation has been widely used (Sharpeshkar, 2010). Owing to its long history compared with other stimulation modalities, electrical stimulation has been trimmed and many solutions for various problems such as charge balancing, stimulation safety, and energy-efficiency of stimulation devices have already been suggested (Aquilina, 2006; Zeng et al., 2008). However, wide coverage of electrical stimulation can also serve as an inherent disadvantage on specificity. To improve the specificity of electrical stimulation, differential stimulation with two closely located electrodes is considered since it can decrease incidence area of stimulation (Ha et al., 2016). A directional electrode scheme is also a good solution for specificity. It modifies orientations of stimulus and restricts areas affected by stimulation so that affected areas are more concentrated on the target area other than non-targeted areas. Instead of the conventional ring shape electrode (Figure 3A), directional electrodes use unique shapes of electrodes (Figure 3B). The common shape of directional electrodes looks like a ring that has been divided in half or other angles and each segment is used as separate electrodes. Even though it shows promising results in simulation, there are still challenges like complicated control of each electrode to shape the electric field (Steigerwald et al., 2019).

Other modalities such as magnetic field and light are also alternatives for better specificity and selectivity. Magnetic stimulation is based on Faraday's law of induction. Alternating current (AC) flowing through a coil generates time-varying magnetic flux, which can couple to tissue at the stimulation site. Then, the flux induces an eddy current at the stimulation site, which can finally evoke neural activities. By adjusting the shape of the lead, the magnetic field is focused on a specific point, allowing for a few hundred μm spatial resolution (Ryu et al., 2020). Optical stimulation requires genetic modification of specific cell types for the formation of light-sensitive ion channels on the cell types. By modulating (excitation or inhibition) activity of the particular cell types using light via small form factor light sources such as micro-LEDs, optical stimulation can increase cell selectivity (Mickle et al., 2019; Kim et al., 2021).

### 3.2. Principle of the Electric Stimulation

When an electrical stimulus is injected via an electrode, the potential of the electrode, and thus the electric field around the electrode, is changed. While charge distribution is modified by the electric field, ionic flows are created across the cell membrane. Then membrane potential deviates from its original state. If the strength and duration of the stimulus are larger enough to incur membrane potential going beyond a threshold voltage for sodium influx, an action potential occurs (Plonsey and Barr, 2007). The shorter the stimulation time and the longer the distance between the electrode and the target neuron are, the larger the stimulation strength and the longer the duration are required. The minimal magnitude of the current occurring action potential for an infinite duration is called rheobase. It varies depending on cell type, maturity, and geometric condition between a cell and an electrode (Geddes and Bourland, 1985; Kinnischtzke et al., 2012). Therefore, stimulation strength and pulse duration need to be adjusted. In addition to strength and duration, there are other parameters such as stimulus waveform and frequency that affect stimulation performance (Sahin and Tie, 2007; Wongsarnpigoon et al., 2010). Typical parameter ranges in DBS for movement disorder patients are 2–4 V in amplitude, 60–450 μs in pulse width, 130–185 Hz in frequency, and biphasic square wave. Please note that these parameters should be optimized for a specific patient before application (Butson and McIntyre, 2005; Ramasubbu et al., 2018).

The optimal parameter settings are still unresolved issues. In terms of frequency, superiority between high frequency and low frequency stimulation effects are still debated. Some studies show that higher frequencies are better to suppress epilepsy (Boëx et al., 2007; Yu et al., 2018), while another study shows the opposite (Wang et al., 2016), and a study even suggests that the two methods do not show statistically significant differences (Wongsarnpigoon et al., 2010. In terms of waveform, other than square waveform, ramp or exponential waveforms are also studied. One study shows that, with the same amount of charge, exponential decaying waveform can activate the largest number of neurons among other waveforms (Lee et al., 2014). However, other studies show different results (Merrill et al., 2005; Wongsarnpigoon et al., 2010).

Besides the efficacy, a stimulus waveform is restricted for safety. Continuous direct current (DC) injects a large net charge into tissue and thus induces electrochemical reactions that can cause permanent tissue damage. Traditionally, the known charge density limit was 30 μ*C*/*cm*^2^ for brain stimulation (Kuncel and Grill, 2004). However, this limit varies based on stimulation parameters such as distance between an electrode and target tissue, pulse frequency, duration, and waveform (Cogan et al., 2016).

Mono-phasic stimulation has no mechanism to actively reverse electrochemical reactions that occurred during the stimulation period. Therefore, its charge injection limit to prevent damage is much smaller than bi-phasic stimulation. Stimulation using waveform of exponential shape shows low damage owing to its fast recovery (Merrill et al., 2005).

**Figure 4:**
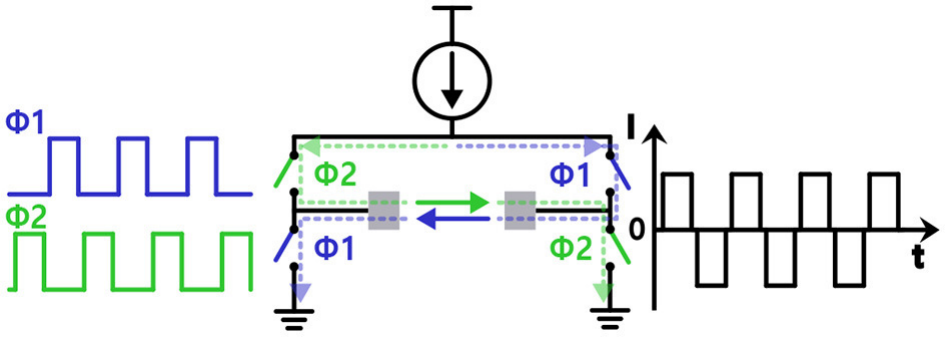
Injecting biphasic current stimulation with a single current source using a H-bridge structure.

Since biphasic waveform is superior to charge balancing and thus is better in terms of tissue damage compared with monophasic waveform, the devices usually apply alternating current (AC) stimulus to avoid net charge flowing into tissue (Merrill et al., 2005; Lee et al., 2013). However, there can be a net current flow due to a mismatch in sourcing and sinking current. Some studies use triphasic or even higher phases stimulus to actively adjust the net charge to zero (Nam et al., 2009; Chu et al., 2013). They detect the mismatch in charge and additionally insert a small stimulus in the opposite direction. Other studies suggest using one single current source to ultimately minimize current mismatch with an H-bridge switch matrix shown in Figure 4 (Sharpeshkar, 2010). H-bridge circuit makes it possible to supply current in both directions with a circuitry, and thereby in theory, there is no charge mismatch (Zhou et al., 2019). However, in practice, the amount of current provided by a current source always varies depending on a voltage drop across the current source.

### 3.3. Electrical Stimulation Methods

**Figure 5:**
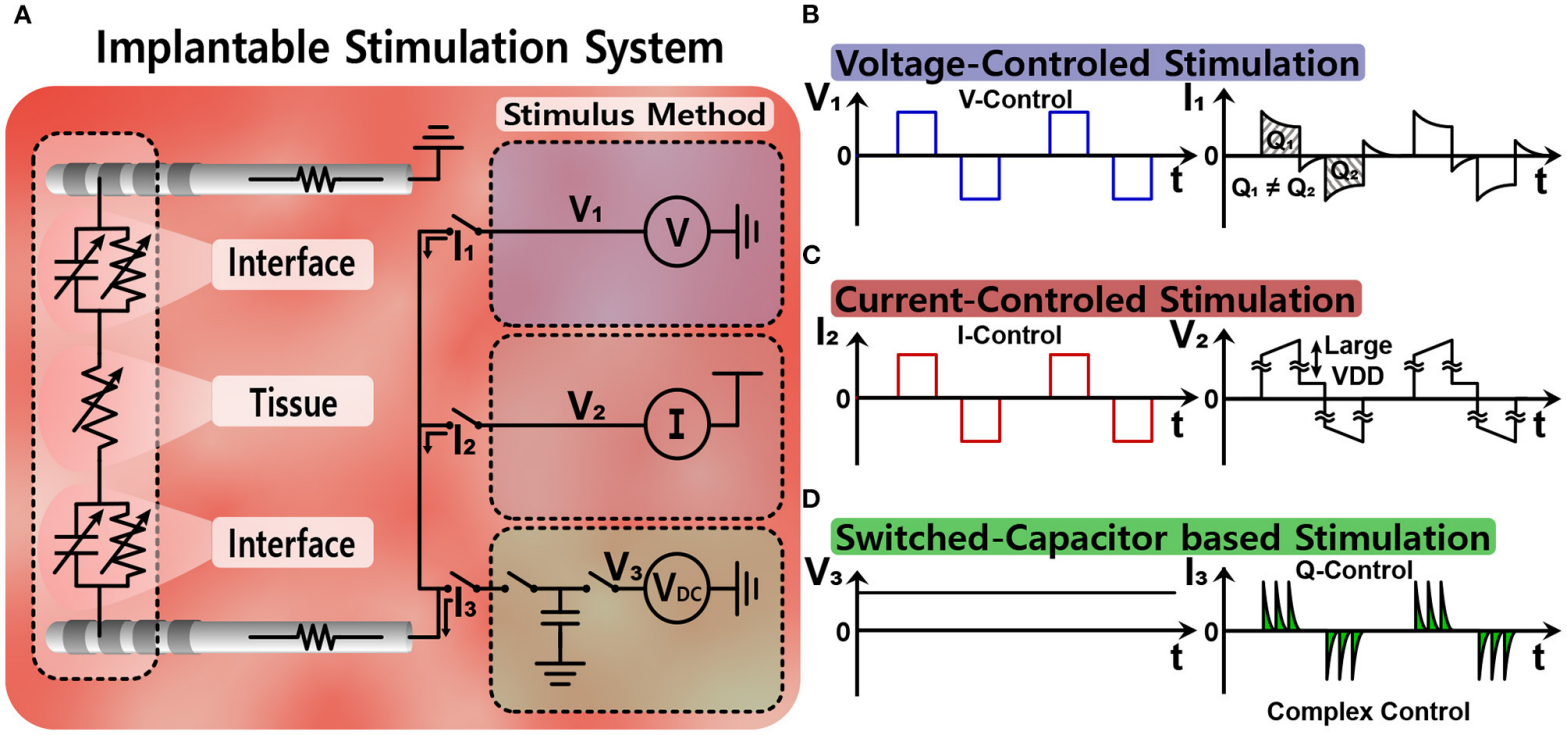
Simple equivalent circuit models of various stimulation methods: **(A)** equivalent circuit models for stimulation, **(B)** voltage-controlled stimulation, **(C)** current-controlled stimulation, and **(D)** switched-capacitor based stimulation.

Figure 5A illustrates three common stimulation methods (Simpson and Ghovanloo, 2007). Voltage-controlled stimulation (VCS) in Figure 5B is a simple and power-efficient structure (Wong et al., 2004). However, the injected charge is likely to be unbalanced because impedance of the electrode-tissue interface varies (Vidal and Ghovanloo, 2010; Lee et al., 2014). Current-controlled stimulation (CCS) in Figure 5C uses current sources instead, and therefore, accurately manages the amount of current for charge balance (Ha et al., 2018). For a high impedance electrode-tissue interface, however, large supply voltage is essential for a sufficient voltage headroom of the current source and it results in high power consumption (Ghovanloo, 2006). Finally, switched-capacitor based stimulation (SCS) in Figure 5D uses multiple capacitors and balances the charge by charging and discharging those capacitors' energy efficiently (Ghovanloo, 2006; Lee et al., 2014). By modifying a DC voltage (V_DC_) and the number of capacitors, the amount of charge injected can be easily adjusted even on the low supply voltage. However, circuit implementation typically becomes more complicated than other methods and its high energy-efficiency cannot be always guaranteed due to power consumption of multiple switches and control circuits. Furthermore, the SCS method has an inherent limitation in that it can generate only exponential waveform from capacitive charging and discharging. Since different waveforms would result in better outcomes depending on patients, a combination of SCS with other methods may be considered.

### 3.4. Open-Loop and Closed-Loop Stimulation

**Figure 6:**
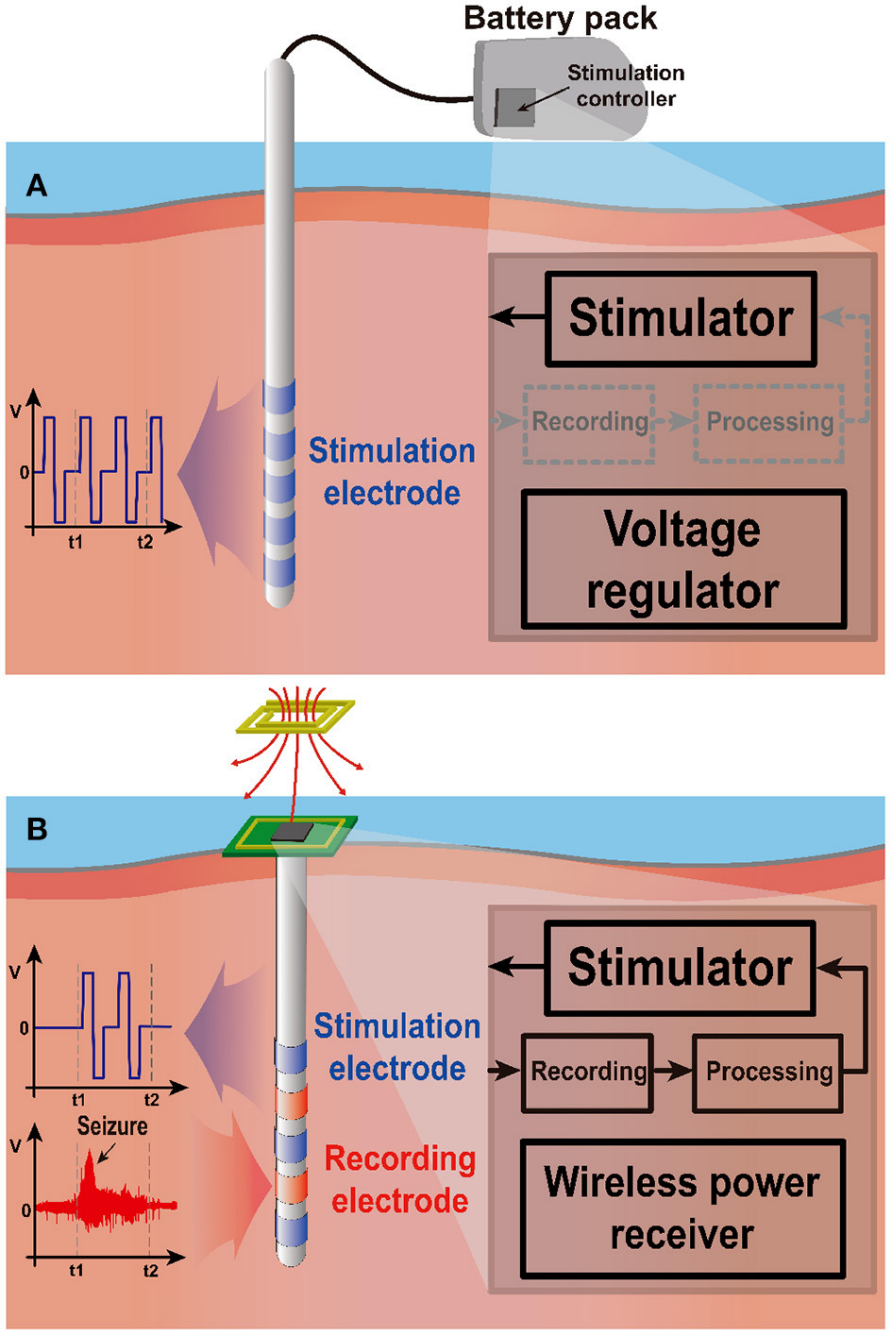
Two stimulation schemes: open-loop and closed-loop stimulation. **(A)** The open-loop system stimulates continuously regardless of the neural activity, requiring large battery pack outside of the stimulation site due to high power consumption. **(B)** The closed-loop system stimulates only when needed, leading to low power consumption. Wireless power transmission can power the implanted stimulation system.

Figure 6A depicts the open-loop stimulation system performing continuous preset stimulation regardless of a subject's current state. This has been commonly used in DBS applications to relieve tremor symptoms of PD patients (Bouthour et al., 2019). Since open-loop stimulation systems do not include monitoring function, medical specialists should regularly check the condition of a subject and adjust stimulation parameters. Persistent stimulation can also increase the risk of side effects (Rosin et al., 2011; Vassileva et al., 2018). Moreover, it requires a large amount of power compared with closed-loop stimulation (Little et al., 2013). Thus, a large battery pack is typically required.

On the other hand, closed-loop stimulation shown in Figure 6B continuously monitors a subject's conditions to determine whether stimulation is needed. Therefore, the stimulation device can provide responsive neural stimulation. In this way, closed-loop stimulation minimizes side effects due to overstimulation and improves power efficiency by preventing unnecessary stimulation. A study shows that although the current consumption of closed-loop configuration is a bit higher during stimulation 230 μA compared with that of open-loop configuration 220 μA, a system in the closed-loop configuration draws only 60 μA from supply for continuous monitoring during the non-stimulation period such that it significantly increases battery operation time (Khanna et al., 2015). A different study also reports that a closed-loop stimulation system improves power consumption by 331 times compared with an open-loop case for identical seizure inhibitory performance (Salam et al., 2015). Thanks to its lower power consumption, the closed-loop system can be powered by a wireless power transfer (WPT) system. This enables further miniaturization by eliminating battery entirely.

It is important to accurately detect bio-markers that indicate a precursor symptom. If the system fails to capture bio-markers, it results in no or delayed stimulation and decreases treatment effects. Therefore, the system requires the following conditions: First, a monitoring device should record neural activities without distortion under severe stimulation artifact. Second, the artifact should be eliminated without loss of neural information so that the bio-makers can be detected correctly even during stimulation. Finally, all building blocks including monitoring parts must operate at low power due to the limited power delivery of WPT.

## 4. Design of Neural Recording Circuits for Closed-Loop Stimulation

Closed-loop stimulation systems require neural recording circuits to figure out appropriate timings for stimulation. The design of the recording circuits is more challenging than conventional neural recording circuits due to the existence of the stimulation artifact. In this section, proper architectures for the closed-loop neural recording are studied.

### 4.1. Requirements for Recording Circuits

**Figure 7:**
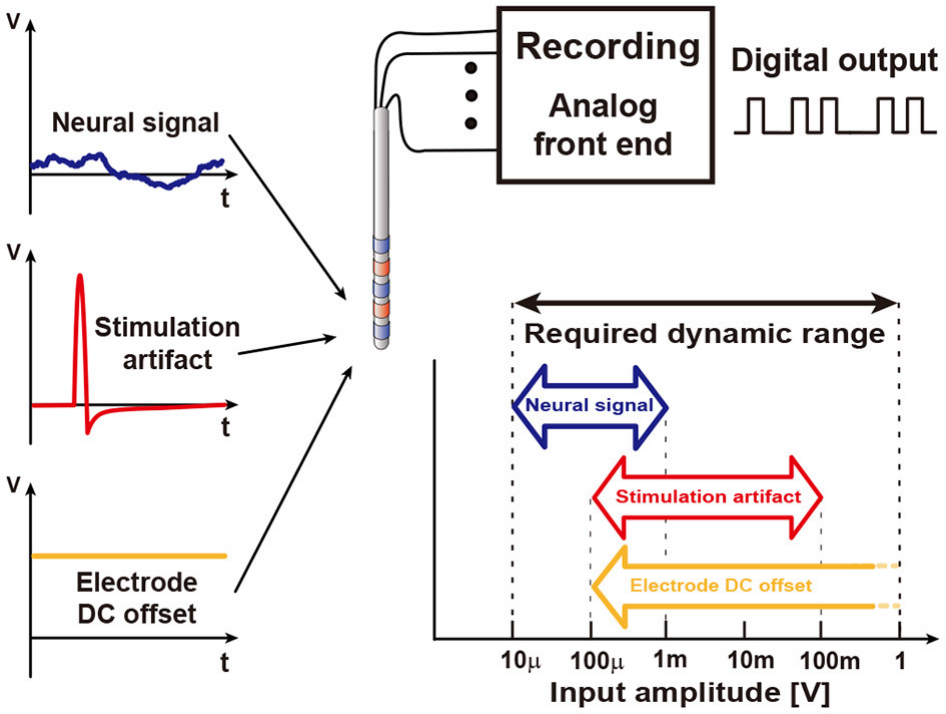
Various inputs to a recording circuit. The amplitudes of these inputs are broad, forcing the recording circuit to have wide dynamic range.

The main purpose of recording circuits is to read analog neural signals via electrodes and to perform analog to digital conversion. Figure 7 illustrates typical incoming signals to an analog front end (AFE). While neural signal ranges in 10 μV–1 mV, other inputs such as stimulation artifacts and electrode DC offset are much greater than neural signal. Thus, several design challenges exist for accurate measurement. Required dynamic range is >100 dB for all incoming inputs without distortion. High dynamic range typically demands high power consumption and large area of the recording circuit. To alleviate dynamic range requirement, researchers should first tackle electrode DC offset (EDO). EDO comes from a half cell potential of electrodes (Weast, 1974; Franks et al., 2005; Ashby and Jones, 2012). The half-cell potential is a function of the electrode materials (e.g., AgCl: 223 mV, Pt: 758 mV, Au: 1.68 V). Differential recording with the same electrodes (at least the material of the electrode) reduces EDO to <100 mV (Denison et al., 2007; Yazicioglu et al., 2007). In addition, EDO can be rejected by adopting an AC coupling method (Harrison and Charles, 2003; Verma et al., 2009; Ng and Xu, 2013) along with a DC servo loop (Kassiri et al., 2016; Luo et al., 2018) at the expense of low frequency components in the neural signal. Even if EDO is fully rejected, >80 dB dynamic range is required under stimulation conditions.

Low input referenced noise (IRN) is essential for an acceptable signal-to-noise ratio (SNR, typically ≥ 10 dB) since the amplitude of the neural signal is a couple of hundred μV. Low power AFE typically becomes the main noise source. In addition to noise, signal attenuation should be minimized by having >100 MΩ input impedance. Large input impedance also reduces DC input current, which otherwise may cause electrochemical reaction and cell damage (Harrison and Charles, 2003; Merrill et al., 2005; Jochum et al., 2009). Multi-channel recording adds a severe restriction on power consumption. Table 1 summarizes design requirements.

**Table 1:** Recording system requirements.

**Specifications**	**Target value**
DC offset	<100 *mV*
Input range	50–100 *mV*
Input referred noise (1–200 Hz)	<10 *μVrms*
Dynamic range	>80 *dB*
DC input impedance	>100 *MΩ*
Power consumption	<10 *μW/ch*

### 4.2. Neural Recording With Amplification

**Figure 8:**
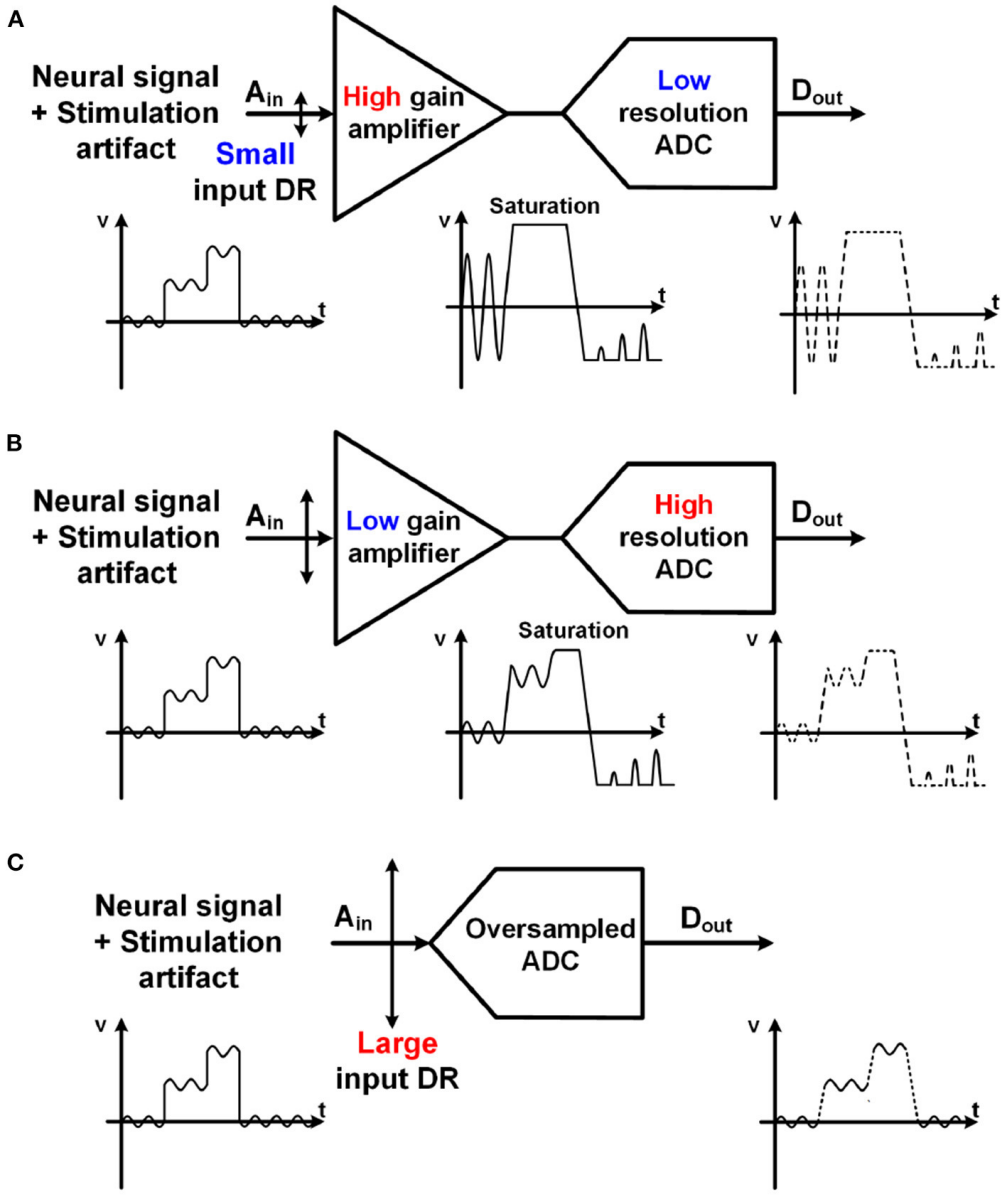
Comparison with various structures for neural recording with stimulation artifact. Both **(A)** a high gain amplifier with a lower resolution ADC and **(B)** a lower gain amplifier with a higher resolution ADC have potential for saturation due to large stimulation artifact. **(C)** Unity gain ADC-direct front-end structure using an oversampling ADC maximizes its input range.

Quantization noise (Q-noise) is the inherent noise of an analog-digital converter (ADC), and a major noise source of neural recording. With a high gain amplifier, a small neural signal is amplified to several hundred millivolts to overcome the quantization noise (Harrison and Charles, 2003; Gao et al., 2012; Han et al., 2013; Lee S. et al., 2018). Meanwhile, for low power implementation, supply voltage for a neural amplifier has been extremely lowered to even 0.2 V (Yaul and Chandrakasan, 2016). Due to these two conditions, high gain and low supply voltage, input ranges of the neural amplifiers are severely limited because input signal of the amplifier cannot be greater than a supply voltage divided by the amplifier's voltage gain. This is why this architecture cannot be considered for neural recording in closed-loop neural interfaces, which have large stimulation artifacts as shown in Figure 8A.

As an alternative, a structure using a lower gain amplifier and a high-resolution ADC in Figure 8B has recently been utilized (Chandrakumar and Marković, 2017, 2018). The gain of the amplifiers is lower than 10. As such, the input range of the neural recording is improved even with a low supply voltage. However, it still has a potential for signal saturation when a large stimulation artifact exists. Furthermore, the benefits from a low gain amplifier are offset by disadvantages such as the area and power consumption for the amplifier.

### 4.3. ADC-Direct Front-End

**Figure 9:**
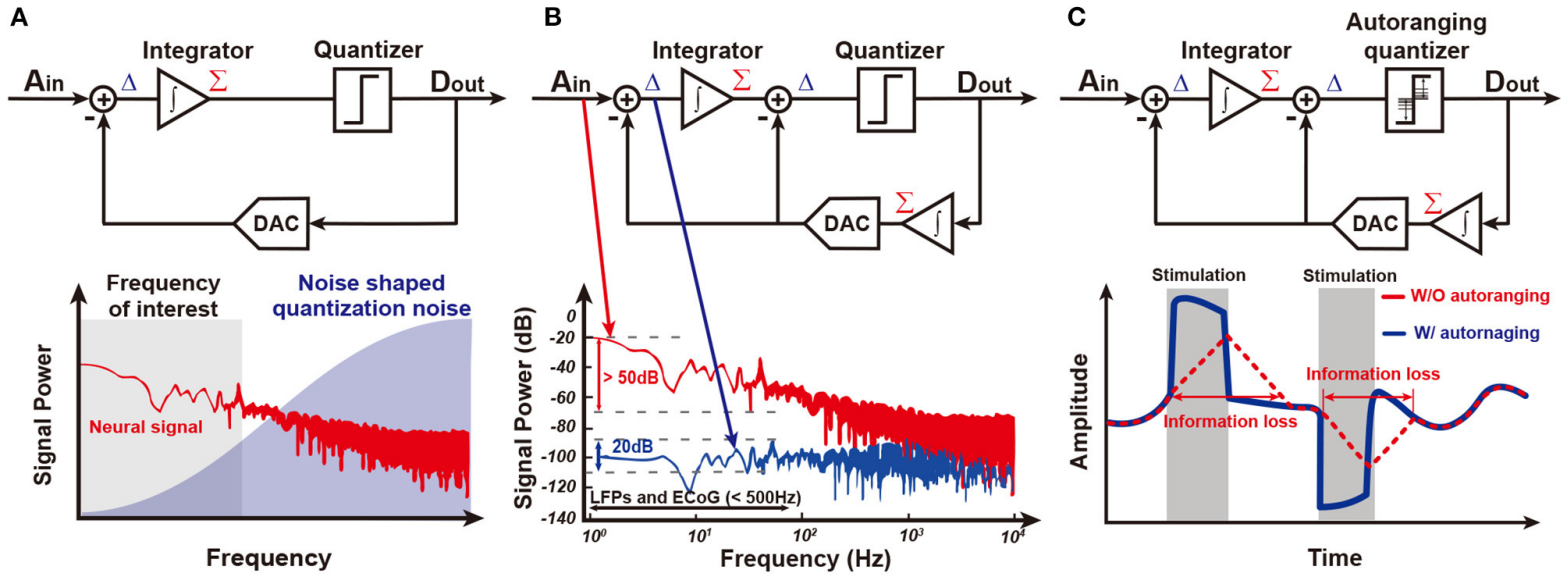
Several ΔΣ structure. **(A)** ΔΣ shapes quantization noise into high frequency. **(B)** An integrator in feedback path of ΔΣADC reduces the required input integrator's dynamic range. This structure is called Δ^2^ΣADC (Kassiri et al., 2017). **(C)** An auto-ranging quantizer in Δ^2^Σ makes it possible to follow sudden changes in the input signal (Kim et al., 2018).

Figure 8C shows an ADC-direct front-end structure. It directly converts analog input to digital output without preamplification, leading to an area and power saving effect. Since there is no amplification, the quantization noise (Q-noise) of ADC is directly compared with the small neural signal at input, and thus it is essential for the Q-noise of ADC to be smaller than neural signal. Although a conventional Nyquist sampling rate ADC including a successive-approximation (SAR) ADC may achieve this low Q-noise, an oversampling delta-sigma ADC (ΔΣADC) is much more energy-efficient to obtain low in-band Q-noise owing to its noise-shaping characteristic. This is why most recent neural recording systems for closed-loop neural interfaces are implemented with noise-shaped delta-sigma ADCs. The oversampling technique utilizes a higher sampling frequency, *f*_*s*_, than needed and thus reduces the Q-noise in-band because the Q-noise is uniformly spread out over ±*f*_*s*_/2. To further decrease the noise, loop filters are added in ΔΣADC. As such, in-band Q-noise is decreased by 3 dB, 9 dB, and 15 dB for every doubling of sampling frequency when zero-order, first-order, and second-order loop filters (integrators) are utilized, respectively. A block diagram for the first-order ΔΣADC with its spectrum of signal and Q-noise are shown in Figure 9A. Neural signal has 1/*f*^*N*^ (2≤N≤3) (Reza Pazhouhandeh et al., 2020) low-pass profile in spectrum while in-band Q-noise is minimized owing to noise shaping (Schreier et al., 2005). In addition to low Q-noise, continuous ΔΣADC includes an inherent anti-aliasing filter. This is because an integrator in ΔΣADC performs low-pass filtering before sampling (Pavan et al., 2008).

However, a ΔΣADC is able to be further improved in the input of dynamic range and power consumption by adding an additional integration function in the feedback path as shown in Figure 9B. The integrator in the feedback path accurately predicts input signal, *A*_*in*_. Delta function (subtraction) between *A*_*in*_ and an estimate from the integrator enable consecutive difference processing. This resolves a famous problem of conventional ΔΣADC, limit cycle, and minimizes the input signal to the integrator in main path by around 30 dB (Kassiri et al., 2017; Bang et al., 2018; Kim et al., 2018; Reza Pazhouhandeh et al., 2020). Furthermore, the integrator in the main path can be implemented by a low-power open-loop Gm-C filter owing to its small input, allowing for drastic power reduction. This structure is named Δ^2^ΣADC.

Although the integrator in the feedback path presents many great advantages, it slows down the speed of prediction. Since updates from the quantizer are averaged by the integrator, prediction speed is limited. This is a serious problem in closed-loop stimulation since the stimulation artifact is large and there is steep variation in amplitude. Actually, utilizing history of the quantizer outputs, ADC detects whether it is slower than input by itself (Kim et al., 2018). If several consecutive outputs of the quantizer, *D*_*out*_, have an equal sign, an ADC needs to tract its input faster, leading to an increase in the update size in the prediction. On the other hand, if consecutive output of the quantizer is alternating, this ADC reaches its input and needs to decrease the update size. This autoranging algorithm improves prediction speed up to 30 times in bandwidth and results in faster than 200 mV/ms tracking speed as shown in Figure 9C.

While complementary metal-oxide-semiconductor (CMOS) technology scaling improves the area, speed, and power efficiency of circuitry, the design difficulty of analog circuits becomes more challenging due to the lowered supply voltage of the scaled devices. Thus, rather than using voltage, time or frequency can be a more useful source with modern CMOS devices. A voltage-controlled oscillator (VCO) based ADC that converts input voltage signal to the frequency domain by VCO and processes the converted signal rather than the voltage domain is one of the great examples (Muller et al., 2012, 2015; Huang and Mercier, 2020). Similar to DSM ADC, a VCO-based ADC structure is also implemented as the ADC-Direct front-end structure.

Table 2 shows a comparison of state-of-the-art neural recording AFEs. It is clearly visible that a conventional large gain amplifier with a separate ADC structure has limited input range such that it is difficult to be applied to closed-loop systems due to huge stimulation artifacts. Furthermore, ADC-direct front-ends have better input referred noise performance while consuming lower power and taking up a smaller area.

**Table 2:** Metric comparison with state-of-the-art.

	**JSSC'16**	**JSSC'18**	**JSSC'17**	**JSSC'18**	**JSSC'20**	**JSSC'20**
	**Kassiri**	**Chan**	**Kassiri**	**Kim**	**Reza**	**Huang**
Structure	High gain + ADC	Low gain + ADC	Δ^2^∑	Δ^2^∑	Δ^2^∑	VCO based
			ADC-direct	ADC-direct	ADC-direct	ADC-direct
Input range (mV_pp_)	4	200	Rail-to-rail	260	1,000	250
IRN (nV/√Hz)	133	127	101	44	71	53
Zin (MΩ)	Gate input	1520	1	26	1,465	4
SNDR (dB)	44.5	86	72.2	66.17	69.8	89.2
Power (μW/ch)	9.1	7.3	0.63	0.8	1.7	3.2
Area (mm^2^/ch)	0.09	0.113	0.013	0.024	0.023	0.08
NEF	6.9	15.3	2.86	1.81	2.86	4.06
FOMs^*^	132	160	161	154	155	171

* *FOMs: SNDR+ 10log(BW/P)*.

## 5. Real-Time Stimulation Artifact Removal

A stimulation artifact typically has a large amplitude and may overlap with the neural signal in spectrum, contaminating neural recording (Zhou et al., 2018). Thus, the stimulation artifact distorts the results of signal processing with contaminated data and leads to the failure of stimulation to be triggered on time (Hartmann et al., 2014). Therefore, real-time stimulation artifact removal is essential for closed-loop stimulation systems.

### 5.1. Stimulation On-Off Timing Decision

**Figure 10:**
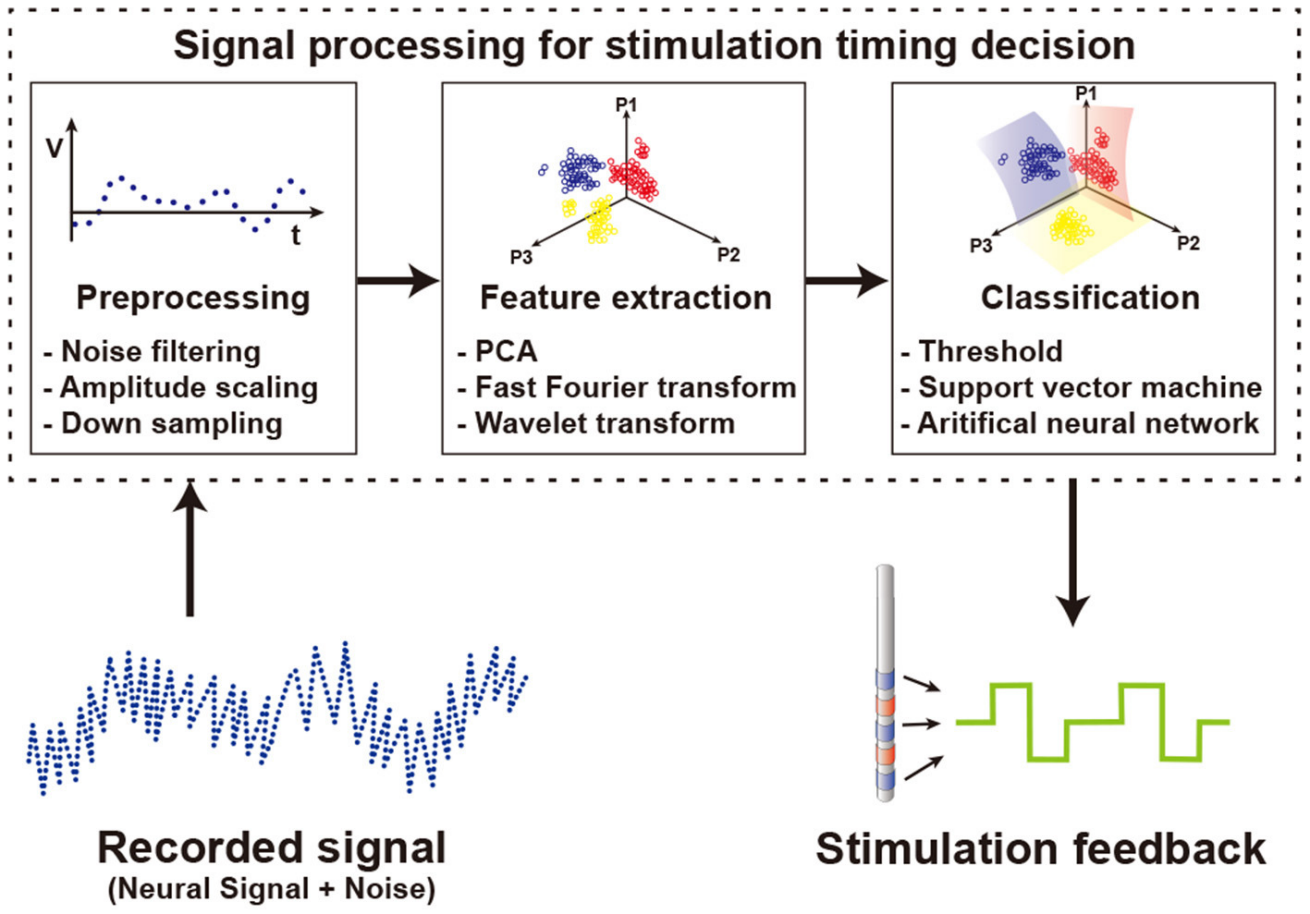
Signal processing to determine the timing of the stimulation using the recorded neural signals.

To determine the stimulation on-off timing, a recorded signal is processed as shown in Figure 10. First, the recorded signal goes through a preprocessing step. In the input data, there are not only target signals but also additional unwanted signals such as power noise, baseline drift, stimulation artifacts, and non-targeted biological signals included. To remove undesired data, conventional spectral filters including notch filter, high pass filter, or bandpass filter are used. Not only do the recorded data differ in the amplitude of the signal for each patient, but a large amount of data makes it difficult to process the signal. To solve these problems, the input data is normalized by amplitude scaling and the size of the data is reduced by downsampling.

Then, features of the preprocessed data are extracted by using several methods such as principal components analysis (PCA), fast Fourier transform (FFT), and wavelet transform (WT). PCA is used to find orthogonal vectors that best represent the variance of input data. By selecting the top several principal component vectors and projecting data onto them, the number of features is highly reduced while minimizing the loss of variance of data. An FFT scheme extracts features of data in frequency domain. Since specific spectral band power varies based on brain activities, spectral power information is a commonly used feature in biosignal processing. WT uses a special set of functions called wavelet to decompose signals. While FFT produces features having only frequency domain information, WT produces features including both time and frequency domain information. Generally, a feature set that combines information from various domains shows better performance than using multiple features from a single domain (Mormann et al., 2003; Kuhlmann et al., 2010). At the first glance, an increase in the number of features brings more information, as such classification performance may be better. Unfortunately, however, this is not always the case. A large number of features for classification increase computational loads and typically decrease overall performance. Therefore, it is important to find the most informative feature set. In the case of neural signals, the characteristics of signals vary from patient to patient. Therefore, selecting features optimized for an individual patient shows better performance than using an identical feature set regardless of the patient (Gadhoumi et al., 2013). To find optimal feature sets supporting patient variation and thus ultimately improve seizure classification performance, a study extracts features from temporal, spectral, and spatial domains (Yoo et al., 2013). Finally, the classification process determines whether to stimulate based on the selected feature set. Threshold based classifiers separate classes if the feature value crosses the threshold (Abdelhalim et al., 2013; Zhou et al., 2019). Since each patient shows unique signal patterns for similar symptoms (Bin Altaf and Yoo, 2016), it is difficult to find a universal optimal threshold value. Therefore, studies adjust and optimize the threshold value for each individual patient after several trials. Other classifiers use machine learning schemes for classification. The support vector machine (SVM), one of the most popular techniques for classification, is a method that maximizes the distance between decision boundary and data in the vicinity of the boundary. Depending on the type of kernel used in the SVM, linear SVM or nonlinear SVM is implemented (Yoo et al., 2013; Bin Altaf and Yoo, 2016). Other than SVM, using artificial neural networks (ANN) as a classifier is also on the rise (Fang et al., 2019; Sayeed et al., 2019). ANN performs classification by connecting nodes at different layers with random weights and optimizing these weights. Though this optimization process can extract optimized features for each patient from raw data without previous feature extraction and selection processes (Alom et al., 2019). However, classifiers with complex algorithms requiring higher computational loads do not always guarantee better results (Kassiri et al., 2017). From a hardware perspective, more complex algorithms require more power and more area. Therefore, based on the purpose of use, an appropriate classifier should be chosen. A study comparing various classifiers for seizure detection shows that area, dynamic power consumption, and signal processing latency are highly different between algorithms. Simple algorithms such as logarithm regression or naive Bayes consume a smaller area and less power than complex algorithms such as SVM or ANN (Page et al., 2014).

### 5.2. Origin of Stimulation Artifact

Stimulation artifacts can be divided into direct stimulation artifact and residual stimulation artifact based on their cause (Zhou et al., 2018). A direct stimulation artifact is caused by stimulation pulses directly reaching neural recording front-ends. Thus, it is large in amplitude and lasts for the stimulation duration. The waveform of the direct stimulation artifact is not the same as that of the stimulation because there is non-linear parasitic capacitance and resistance between a stimulation electrode and a recording electrode. This makes it difficult to predict stimulation artifacts accurately at neural recording front-ends.

A residual stimulation artifact is created by a residual charge left in double-layer capacitance induced by the stimulation electrode after stimulation. This residual charge contaminates tissue potentials in the vicinity of the stimulation electrode, and thus it is considered as “an artifact.” The main causes of this residual charge are (1) current mismatching between the sourcing and sinking current of the stimulator, and (2) electrical characteristic non-linearity of the stimulation electrode during stimulation. The decaying time constant of this residual charge is directly related to the double-layer capacitance and tissue resistance. The typical amplitude of a residual stimulation artifact is a couple of millivolts (Zhou et al., 2018), which is still larger than a neural signal. Therefore, depending on electrode design, a residual stimulation artifact can be long-lasting, and become more serious than a direct stimulation artifact (Hashimoto et al., 2002; Chu et al., 2013).

### 5.3. Stimulation Artifact Removal Methods

**Figure 11:**
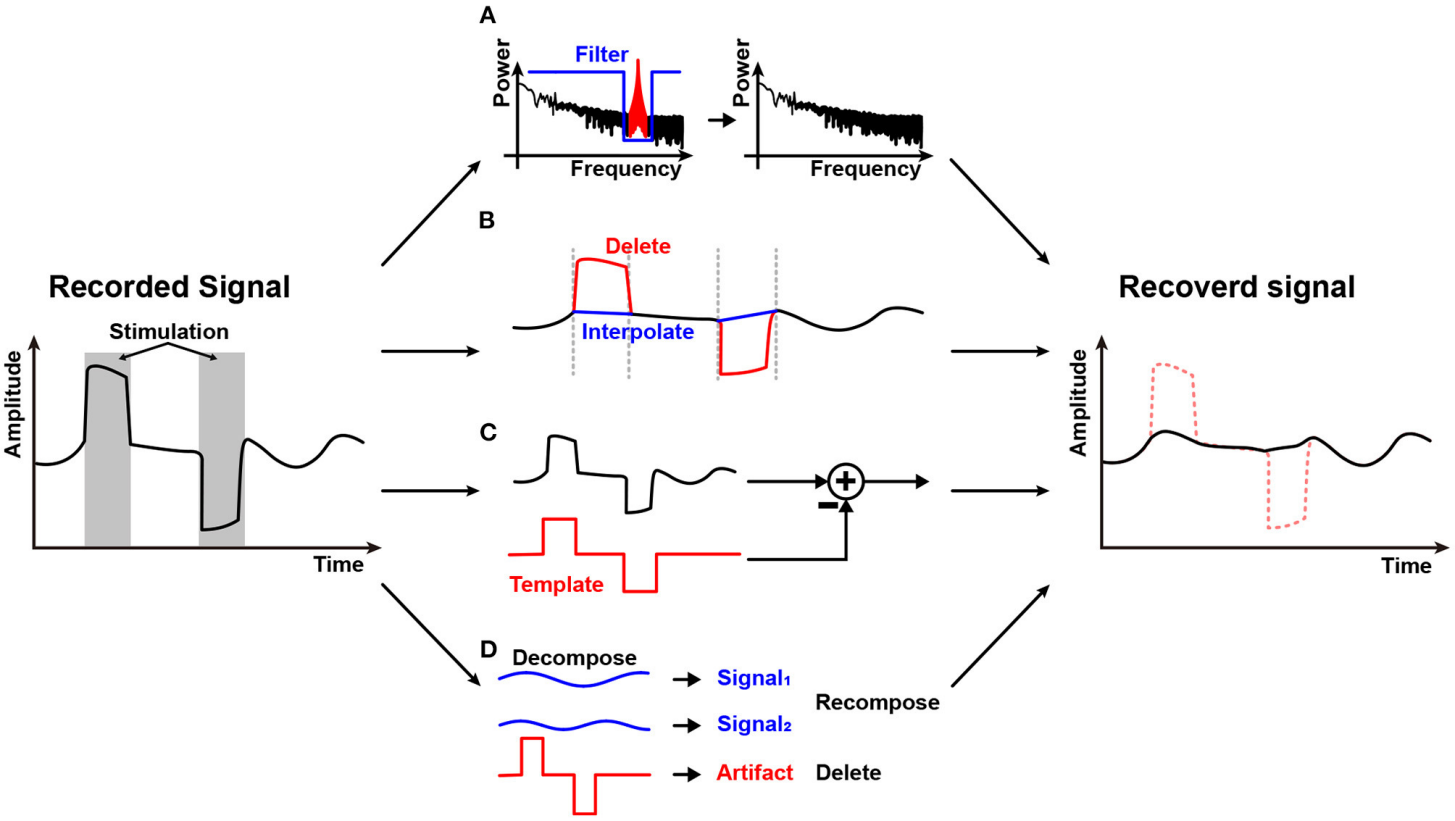
Stimulation artifact removal methods: **(A)** Spectral filtering; **(B)** Blanking and interpolation; **(C)** Template-based subtraction; and **(D)** Components decomposition.

Stimulation artifact removal is important for implementing closed-loop stimulation systems. The first step to reduce a stimulation artifact is to utilize differential recording for a direct stimulation artifact and to adopt charge balancing techniques for a residual stimulation artifact. However, these schemes cannot remove a stimulation artifact entirely. Therefore, additional processes for stimulation artifact removal have been studied as depicted in Figure 11.

Spectral filtering shown in Figure 11A may be the first method that comes to mind to remove the stimulation artifact. Direct stimulation artifact typically has a strong tone in spectrum. Therefore, spectral filtering may work well (Jech et al., 2006). However, stimulation frequency is an important parameter for stimulation efficacy and as such, it should be adjusted regularly. Furthermore, stimulation and a neural signal may overlap in spectrum with each other (Zhou et al., 2018). In addition, it is difficult to avoid spectral distortion.

Figure 11B shows a blanking and interpolation scheme. Since the stimulation timing is known, it is relatively easy to reject or remove direct stimulation artifact by halting data recording during stimulation or deleting data that contains stimulation artifact (Hartmann et al., 2014; Li et al., 2019). Then, data prediction should be accompanied. Various techniques such as linear (Zhou et al., 2019), cubic spline (Waddell et al., 2009), and gaussian (Caldwell et al., 2020) interpolation are utilized. Depending on the algorithm's complexity, the real-time operation is possible (Zhou et al., 2019). However, interpolation inherently produces artificial data and has a chance to miss abrupt events that may happen during the stimulation period. Therefore, this method is not suitable for stimulation devices that use high-frequency stimulation or have long and unpredictable duration artifacts (Cheng et al., 2017).

Some studies make a template of a stimulation artifact and subtract it in the recording process as shown in Figure 11C. The stimulation artifact template is obtained by using various methods such as adaptive filter (Mouthaan et al., 2016), averaging signals (Qian et al., 2016), curve fitting (Drebitz et al., 2020), and equivalent circuit modeling (Trebaul et al., 2016). The earned stimulation artifact template is then applied to the input of an AFE (Wang et al., 2020) or to digitized signal for subtraction at digital signal processing module (Limnuson et al., 2015). Subtraction at the input of an AFE relieves the dynamic range requirement of the AFE at the expense of input impedance and noise performance. Since the template copies only the stimulation artifact, the original neural signal remains after subtraction. However, this scheme heavily depends on the accuracy of the stimulation artifact estimation.

Component decomposition techniques such as principal component analysis (Chang et al., 2019), independent component analysis (Lu et al., 2012), and ensemble empirical mode decomposition (Zeng et al., 2015) to separate stimulation artifact components from contaminated neural signals are illustrated in Figure 11D. Since decomposition requires heavy computational resources, this scheme is usually conducted in the digital domain, and in a non-realtime fashion with a high-performance digital processor. This technique is possibly applied together with the aforementioned techniques such as a template method (Wang et al., 2020).

### 5.4. Avoidance of Stimulation Artifact

Rather than removing the stimulation artifact, as an alternative, schemes for stimulation artifact avoidance are also studied by using different stimulation modalities such as magnetic field or light (Mickle et al., 2019; Ryu et al., 2020). It seems to be obvious that non-electrical stimulation will not generate any electrical artifacts. Unfortunately, however, this is not always true. Magnetic stimulation is fundamentally an electrical stimulation since this relies on induced current by electromotive force. Therefore, it could distort recorded electric signals. Optical stimulation also possibly induces electrical stimulation artifact when photons hit any obstacles due to photovoltaic effect or photoelectrochemical effect (Liu et al., 2018; Kim et al., 2020). In addition, this requires genetic modification for light-sensitivity ion channel expression on target cells, imposing hurdles upon its application to human subjects.

## 6. Wireless Power Transfer for Miniaturized Implantable Devices

Reducing implantable devices' volume is essential to prevent inflammation, glial scar, and even cell death inside a human body (Anderson, 1988). To diminish the volume of batteries that account for a significant portion of the implantable device is the most effective way to reduce the device's volume. Moreover, eliminating batteries prevents frequent surgeries for battery replacement (Bock et al., 2012). Since the wireless power transfer (WPT) technique enables continuous power delivery to implantable devices wirelessly, miniaturized implantable devices without batteries become feasible. However, the amount of power delivery by WPT schemes is typically lower than that by battery. Therefore, many implantable devices have been operated by batteries at the expense of large volume. By the fact that closed-loop neural interface systems consume lower power owing to on-demand stimulation compared to open-loop systems, researchers have recently been putting significant efforts into employing WPT schemes for power delivery to the implantable closed-loop systems, especially for a closed-loop deep brain and vagus nerve stimulation (Rhew et al., 2014; Ranjandish and Schmid, 2018). As such, WPT schemes become crucial for implantable closed-loop systems.

**Figure 12:**
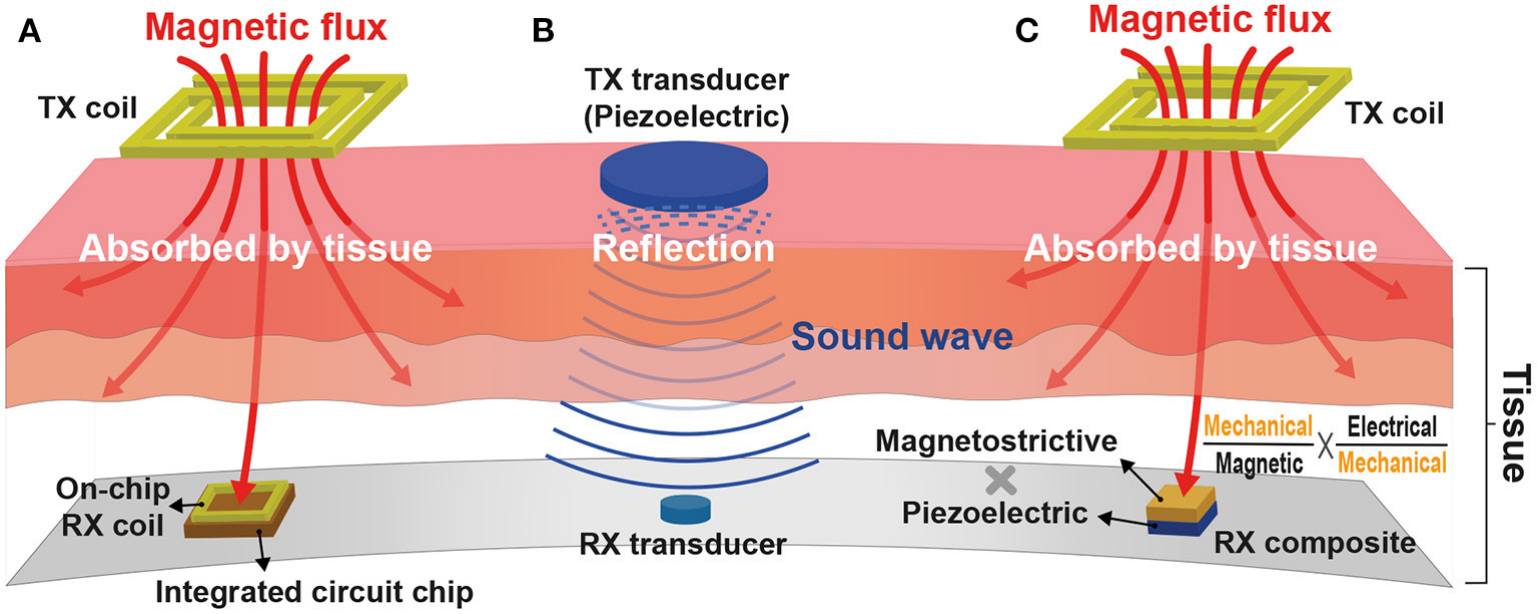
Wireless power transfer (WPT) modalities for a miniaturized implant: **(A)** Inductive WPT using an on-chip power receiving coil; **(B)** Ultrasonic WPT having a short wavelength; and **(C)** Magnetoelectric WPT integrating both inductive and ultrasound WPTs.

Inductive WPT based on Faraday's law of induction is the most historical and steady model among various WPTs. It requires power transmitting (TX) and receiving (RX) coils inductively coupled via induced magnetic flux at the TX coil. The RX coil is typically implemented on a printed circuit board (PCB) for the coil's great quality factor at the expense of implant size and wire connection between the RX coil and a voltage rectifier and a regulator. To achieve ultimate miniaturization of an implant, this RX coil is recently integrated by on-chip, eliminating all off-chip components as shown in Figure 12A (Kim et al., 2016, 2017a,b; Park et al., 2017; Rahmani and Babakhani, 2018a,b). However, the amount of power delivery is directly proportional to RX coil size, and as such, the on-chip RX coil limits the overall amount of power delivered to the implant (≤500μW).

Adopting a higher carrier frequency (≥100 MHz) for power delivery compensates for the reduced amount of power delivery by increasing the magnetic flux variation rate (Kim et al., 2013). Furthermore, a higher carrier frequency allows for a shorter wavelength that provides high spatial-resolution and thus improved WPT energy efficiency (Poon et al., 2010). Unfortunately, however, the tissue absorption rate also rises with the increasing carrier frequency and induces serious safety issues. Considering the fact that sound waves have hundreds of thousands of times shorter wavelengths compared to electromagnetic waves, ultrasonic WPT in Figure 12B would be a perfect alternative against inductive WPT. It consists of two miniaturized transducers that convert sound waves to electricity and vice versa (Charthad et al., 2015). Recent studies successfully demonstrate that mm-sized implantable devices using ultrasonic WPT employed at peripheral nerves are able to receive sufficient power (≤3mW) at a few centimeters implant depth (≤10.5cm) (Charthad et al., 2018; Johnson et al., 2018). However, a high acoustic impedance difference between air, bone, and tissue causes sound wave reflection, limiting ultrasonic WPT applications.

Figure 12C illustrates a newly emerged modality, magnetoelectric (ME) WPT. ME WPT consists of magnetostrictive (MS) and piezoelectric (PE) composite. MS converts magnetic to mechanical, and PE converts mechanical to electrical and vice versa (Nan, 1994; Fiebig, 2005). Such a set of conversions resolves the reflection issue of ultrasound and alleviates the miniaturization difficulty of inductive (Singer et al., 2020; Yu et al., 2020b). Nevertheless, ME composite is hard to fabricate, and the composite's low energy conversion ratio is a hurdle to overcome (Truong, 2020; Yu et al., 2020a).

The detailed performance and specification of the state-of-the-art WPTs are compared in Table 3. It is visible that ultrasound schemes are superior to inductive coupling schemes from a distance, thanks to their lower carrier frequency. Besides, considering the delivered power and distance shown in the table, it is possible to power an implantable closed-loop system consuming lower than a couple of hundred μW (Khanna et al., 2015; Salam et al., 2015). Please note that power efficiency is related to safety issues. Low power efficiency results in high power loss, possibly on tissues, leading to heating problems. If a 1 mm^3^ implant in the brain consumes more than 4 mW, greater than 1°C temperature increases (Kreith and Black, 1980; Giering et al., 1995; Gosalia et al., 2004; Kim et al., 2007).

**Table 3:** Comparison table of the state of the arts WPTs.

	**JSSC'17**	**IMS'18**	**JSSC'15**	**CICC'18**	**TBioCAS'20**
	**Kim**	**Rahmani**	**Charthad**	**Johnson**	**Yu**
Modality	On-chip inductive	On-chip inductive	Ultrasound	Ultrasound	Magnetoelectric
Delivered Power (mW)	0.7	1.2	0.1	0.35	0.09
Power efficiency (%)	0.2	0.1	NR^*^	NR^*^	0.064
Link distance (mm)	10	10	30	21.5	30
Volume (mm^3^)	2.25	0.64	NR^*^	6.5	8.2
Frequency (MHz)	144	434	1	2	0.25
Intervening medium	Air	Air	Muscle	Muscle	PBS

* *NR, Not reported*.

## 7. Conclusion

In this article, current state-of-the-art implantable stimulation devices were reviewed. The advantages and disadvantages of various stimulation modalities, methods, parameters, and control schemes were also studied. Based on these considerations, we highlighted the necessity of closed-loop, miniaturized, low-power designs of implantable devices for the subject's safety and devices' performance. Several crucial requirements for neural recording to implement a closed-loop system include high input dynamic range, fast-tracking, low input-referred noise, high input impedance, and low-power consumption. ΔΣADC direct front-end and VCO-based ADC direct front-end structures are good candidates for those requirements. Problems and causes of stimulation artifact were explained and then several techniques to remove stimulation artifact such as spectral filtering, blanking and interpolation, template-based subtraction, and components decomposition were presented. Finally, representative WPT schemes for eliminating a battery from an implantable device and thus realizing extreme miniaturization were reviewed. In today's era of increasing demand for implantable neuromodulation devices including electroceuticals, a closed-loop neural stimulation device that receives power wirelessly and performs real-time stimulation artifact removal will be an important milestone toward miniaturized neural interfaces.

## Author Contributions

JC, GS, and CK: paper conception and design and drafting of the manuscript and final approval of the manuscript. JC and CK: stimulation applications and neural recording circuits. GS and CK: stimulation system consideration and stimulation artifact removal. YC and CK: wireless power transfer for miniaturized implantable devices. All authors contributed to the article and approved the submitted version.

## Conflict of Interest

The authors declare that the research was conducted in the absence of any commercial or financial relationships that could be construed as a potential conflict of interest.
